# Major Depressive Disorder: Advances in Neuroscience Research and Translational Applications

**DOI:** 10.1007/s12264-021-00638-3

**Published:** 2021-02-13

**Authors:** Zezhi Li, Meihua Ruan, Jun Chen, Yiru Fang

**Affiliations:** 1 Clinical Research Center and Division of Mood Disorders of Shanghai Mental Health Center, Shanghai Jiao Tong University School of Medicine, Shanghai, 200030 China; 2 Department of Neurology, Ren Ji Hospital, Shanghai Jiao Tong University School of Medicine, Shanghai, 200127 China; 3 Shanghai Institute of Nutrition and Health, Shanghai Information Center for Life Sciences, Chinese Academy of Science, Shanghai, 200031 China; 4 Center for Excellence in Brain Science and Intelligence Technology, Chinese Academy of Science, Shanghai, 200031 China; 5 Shanghai Key Laboratory of Psychotic Disorders, Shanghai, 201108 China

**Keywords:** Major depressive disorder, Pathogenesis, Treatment, Progress

## Abstract

Major depressive disorder (MDD), also referred to as depression, is one of the most common psychiatric disorders with a high economic burden. The etiology of depression is still not clear, but it is generally believed that MDD is a multifactorial disease caused by the interaction of social, psychological, and biological aspects. Therefore, there is no exact pathological theory that can independently explain its pathogenesis, involving genetics, neurobiology, and neuroimaging. At present, there are many treatment measures for patients with depression, including drug therapy, psychotherapy, and neuromodulation technology. In recent years, great progress has been made in the development of new antidepressants, some of which have been applied in the clinic. This article mainly reviews the research progress, pathogenesis, and treatment of MDD.

Major depressive disorder (MDD) also referred to as depression, is one of the most severe and common psychiatric disorders across the world. It is characterized by persistent sadness, loss of interest or pleasure, low energy, worse appetite and sleep, and even suicide, disrupting daily activities and psychosocial functions. Depression has an extreme global economic burden and has been listed as the third largest cause of disease burden by the World Health Organization since 2008, and is expected to rank the first by 2030 [1, 2]. In 2016, the Global Burden of Diseases, Injuries, and Risk Factors Study demonstrated that depression caused 34.1 million of the total years lived with disability (YLDs), ranking as the fifth largest cause of YLD [3]. Therefore, the research progress and the clinical application of new discoveries or new technologies are imminent. In this review, we mainly discuss the current situation of research, developments in pathogenesis, and the management of depression.

## Current Situation of Research on Depression

### Analysis of Published Papers

**Fig. 1:**
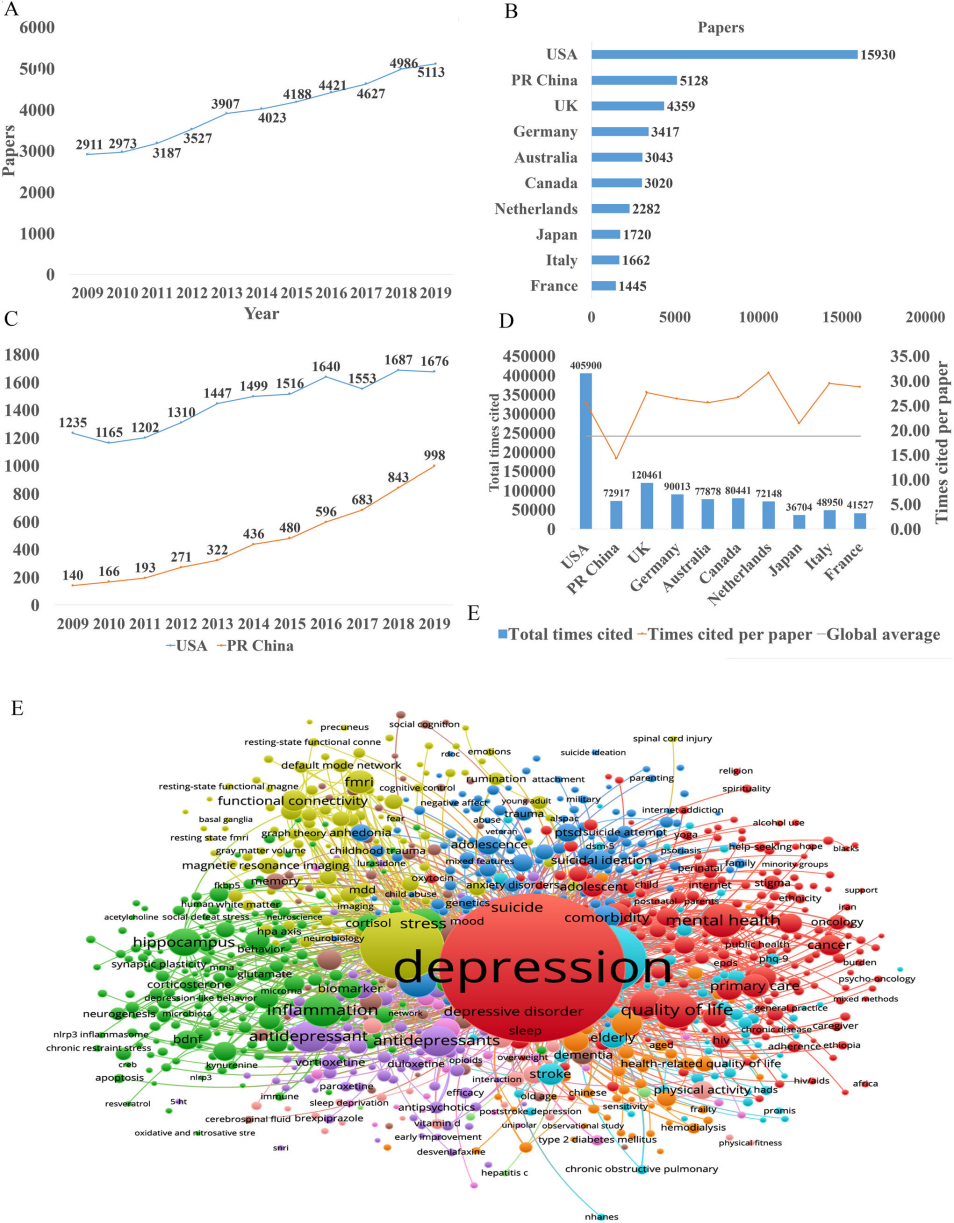
Analysis of published papers around the world from 2009 to 2019 in depressive disorder. **A** The total number of papers [from a search of the Web of Science database (search strategy: TI = (depression$) or ts = ("major depressive disorder$")) and py = (2009*–*2019), Articles)]. **B** The top 10 countries publishing on the topic. **C** Comparison of papers in China and the USA. **D** Citations for the top 10 countries and comparison with the global average. **E** Hot topics.

In the past decade, the total number of papers on depression published worldwide has increased year by year as shown in Fig. 1A. Searching the Web of Science database, we found a total of 43,863 papers published in the field of depression from 2009 to 2019 (search strategy: TI = (depression$) or ts = ("major depressive disorder$")) and py = (2009*–*2019), Articles). The top 10 countries that published papers on the topic of depression are shown in Fig. 1B. Among them, researchers in the USA published the most papers, followed by China. Compared with the USA, the gap in the total number of papers published in China is gradually narrowing (Fig. 1C), but the quality gap reflected by the index (the total number of citations and the number of citations per paper) is still large, and is lower than the global average (Fig. 1D). As shown in Fig. 1E, the hot research topics in depression are as follows: depression management in primary care, interventions to prevent depression, the pathogenesis of depression, comorbidity of depression and other diseases, the risks of depression, neuroimaging studies of depression, and antidepressant treatment.

### Analysis of Patented Technology Application

**Fig. 2:**
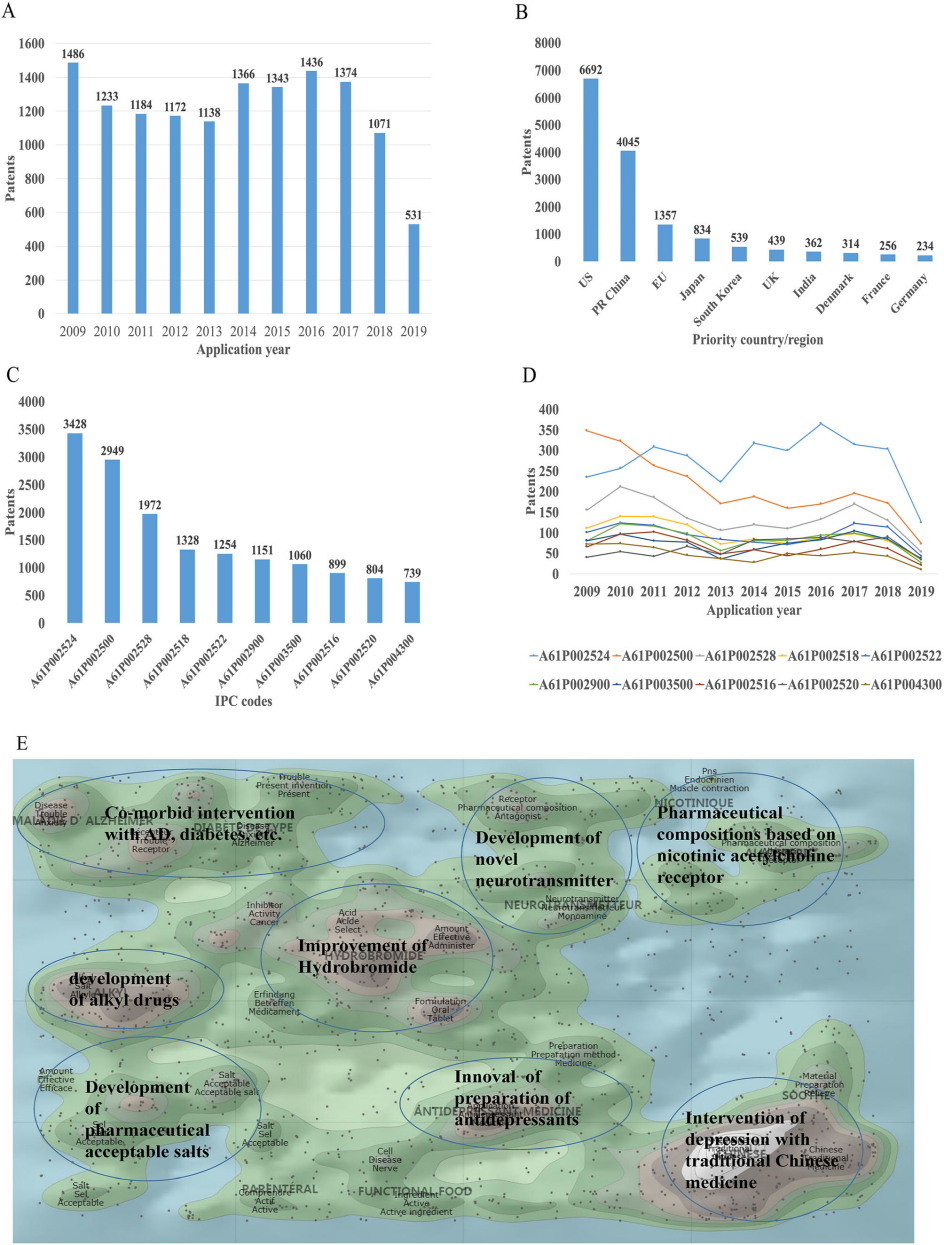
Analysis of patented technology applications from 2009 to 2019 in the field of depressive disorder. **A** Annual numbers and trends of patents (the Derwent Innovation patent database). **B** The top 10 countries/regions applying for patents. **C** The top 10 technological areas of patents. **D** The trend of patent assignees. **E** Global hot topic areas of patents.

There were 16,228 patent applications in the field of depression between 2009 and 2019, according to the Derwent Innovation Patent database. The annual number and trend of these patents are shown in Fig. 2A. The top 10 countries applying for patents related to depression are shown in Fig. 2B. The USA ranks first in the number of depression-related patent applications, followed by China. The largest number of patents related to depression is the development of antidepressants, and drugs for neurodegenerative diseases such as dementia comorbid with depression. The top 10 technological areas of patents related to depression are shown in Fig. 2C, and the trend in these areas have been stable over the past decade (Fig. 2D).

Analysis of technical hotspots based on keyword clustering was conducted from the Derwent Innovation database using the "ThemeScape" tool. This demonstrated that the hot topic areas are as follows (Fig. 2E): (1) improvement for formulation and the efficiency of hydrobromide, as well as optimization of the dosage; intervention for depression comorbid with AD, diabetes, and others; (3) development of alkyl drugs; (4) development of pharmaceutical acceptable salts as antidepressants; (5) innovation of the preparation of antidepressants; (6) development of novel antidepressants based on neurotransmitters; (7) development of compositions based on nicotinic acetylcholine receptors; and (8) intervention for depression with traditional Chinese medicine.

### Analysis of Clinical Trial

There are 6,516 clinical trials in the field of depression in the ClinicalTrials.gov database, and among them, 1,737 valid trials include the ongoing recruitment of subjects, upcoming recruitment of subjects, and ongoing clinical trials. These clinical trials are mainly distributed in the USA (802 trials), Canada (155), China (114), France (93), Germany (66), UK (62), Spain (58), Denmark (41), Sweden (39), and Switzerland (23). The indications for clinical trials include various types of depression, such as minor depression, depression, severe depression, perinatal depression, postpartum depression, and depression comorbid with other psychiatric disorders or physical diseases, such as schizophrenia, epilepsy, stroke, cancer, diabetes, cardiovascular disease, and Parkinson's disease.

Based on the database of the Chinese Clinical Trial Registry website, a total of 143 clinical trials for depression have been carried out in China. According to the type of research, they are mainly interventional and observational studies, as well as a small number of related factor studies, epidemiological studies, and diagnostic trials. The research content involves postpartum, perinatal, senile, and other age groups with clinical diagnosis (imaging diagnosis) and intervention studies (drugs, acupuncture, electrical stimulation, transcranial magnetic stimulation). It also includes intervention studies on depression comorbid with coronary heart disease, diabetes, and heart failure.

### New Medicine Development

**Fig. 3:**
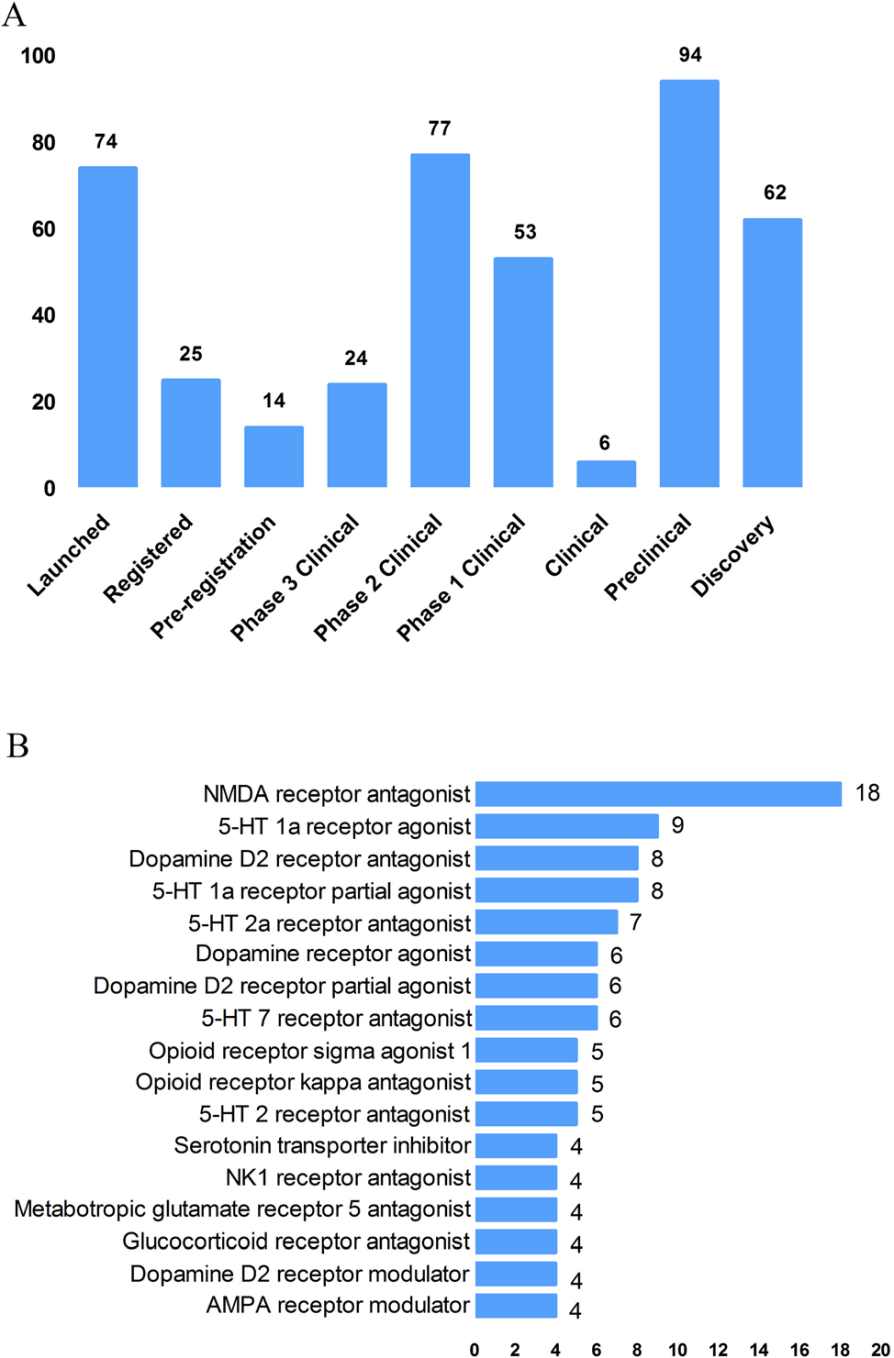
New medicine development from 2009 to 2019 in depressive disorder. **A** Development status of new candidate drugs. **B** Top target-based actions.

According to the Cortellis database, 828 antidepressants were under development by the end of 2019, but only 292 of these are effective and active (Fig. 3A). Large number of them have been discontinued or made no progress, indicating that the development of new drugs in the field of depression is extremely urgent.

From the perspective of target-based actions, the most common new drugs are NMDA receptor antagonists, followed by 5-HT targets, as well as dopamine receptor agonists, opioid receptor antagonists and agonists, AMPA receptor modulators, glucocorticoid receptor antagonists, NK1 receptor antagonists, and serotonin transporter inhibitors (Fig. 3B).

## Epidemiology of Depression

The prevalence of depression varies greatly across cultures and countries. Previous surveys have demonstrated that the 12-month prevalence of depression was 0.3% in the Czech Republic, 10% in the USA, 4.5% in Mexico, and 5.2% in West Germany, and the lifetime prevalence of depression was 1.0% in the Czech Republic, 16.9% in the USA, 8.3% in Canada, and 9.0% in Chile [4, 5]. A recent meta-analysis including 30 Countries showed that lifetime and 12-month prevalence depression were 10.8% and 7.2%, respectively [6]. In China, the lifetime prevalence of depression ranged from 1.6% to 5.5% [7–9]. An epidemiological study demonstrated that depression was the most common mood disorder with a life prevalence of 3.4% and a 12-month prevalence of 2.1% in China [10].

Some studies have also reported the prevalence in specific populations. The National Comorbidity Survey-Adolescent Supplement (NCS-A) survey in the USA showed that the lifetime and 12-month prevalence of depression in adolescents aged 13 to 18 were 11.0% and 7.5%, respectively [11]. A recent meta-analysis demonstrated that lifetime prevalence and 12-month prevalence were 2.8% and 2.3%, respectively, among the elderly population in China [12].

## Neurobiological Pathogenesis of Depressive Disorder

### Monoamines

The early hypothesis of monoamines in the pathophysiology of depression has been accepted by the scientific community. The evidence that monoamine oxidase inhibitors and tricyclic antidepressants promote monoamine neurotransmission supports this theory of depression [13]. So far, selective serotonin reuptake inhibitors and norepinephrine reuptake inhibitors are still the first-line antidepressants. However, there remain 1/3 to 2/3 of depressed patients who do not respond satisfactorily to initial antidepressant treatment, and even as many as 15%–40% do not respond to several pharmacological medicines [14, 15]. Therefore, the underlying pathogenesis of depression is far beyond the simple monoamine mechanism.

Other hypotheses of depression have gradually received increasing attention because of biomarkers for depression and the effects pharmacological treatments, such as the stress-responsive hypothalamic pituitary adrenal (HPA) axis, neuroendocrine systems, the neurotrophic family of growth factors, and neuroinflammation.

### Stress-Responsive HPA Axis

Stress is causative or a contributing factor to depression. Particularly, long-term or chronic stress can lead to dysfunction of the HPA axis and promote the secretion of hormones, including cortisol, adrenocorticotropic hormone, corticotropin-releasing hormone, arginine vasopressin, and vasopressin. About 40%–60% of patients with depression display a disturbed HPA axis, including hypercortisolemia, decreased rhythmicity, and elevated cortisol levels [16, 17]. Mounting evidence has shown that stress-induced abnormality of the HPA axis is associated with depression and cognitive impairment, which is due to the increased secretion of cortisol and the insufficient inhibition of glucocorticoid receptor regulatory feedback [18, 19]. In addition, it has been reported that the increase in cortisol levels is related to the severity of depression, especially in melancholic depression [20, 21]. Further, patients with depression whose HPA axis was not normalized after treatment had a worse clinical response and prognosis [22, 23]. Despite the above promising insights, unfortunately previous studies have shown that treatments regulating the HPA axis, such as glucocorticoid receptor antagonists, do not attenuate the symptoms of depressed patients [24, 25].

### Glutamate Signaling Pathway

Glutamate is the main excitatory neurotransmitter released by synapses in the brain; it is involved in synaptic plasticity, cognitive processes, and reward and emotional processes. Stress can induce presynaptic glutamate secretion by neurons and glutamate strongly binds to ionotropic glutamate receptors (iGluRs) including N-methyl-D-aspartate receptors (NMDARs) and α-amino-3-hydroxy-5-methyl-4-isoxazole-propionic acid receptors (AMPARs) [26] on the postsynaptic membrane to activate downstream signal pathways [27]. Accumulating evidence has suggested that the glutamate system is associated with the incidence of depression. Early studies have shown increased levels of glutamate in the peripheral blood, cerebrospinal fluid, and brain of depressed patients [28, 29], as well as NMDAR subunit disturbance in the brain [30, 31]. Blocking the function of NMDARs has an antidepressant effect and protects hippocampal neurons from morphological abnormalities induced by stress, while antidepressants reduce glutamate secretion and NMDARs [32]. Most importantly, NMDAR antagonists such as ketamine have been reported to have profound and rapid antidepressant effects on both animal models and the core symptoms of depressive patients [33]. On the other hand, ketamine can also increase the AMPAR pathway in hippocampal neurons by up-regulating the AMPA glutamate receptor 1 subunit [34]. Further, the AMPAR pathway may be involved in the mechanism of antidepressant effects. For example, preclinical studies have indicated that AMPAR antagonists might attenuate lithium-induced depressive behavior by increasing the levels of glutamate receptors 1 and 2 in the mouse hippocampus [35].

### Gamma-Aminobutyric Acid (GABA)

Contrary to glutamate, GABA is the main inhibitory neurotransmitter. Although GABA neurons account for only a small proportion compared to glutamate, inhibitory neurotransmission is essential for brain function by balancing excitatory transmission [36]. Number of studies have shown that patients with depression have neurotransmission or functional defects of GABA [37, 38]. Schür *et al*., conducted a meta-analysis of magnetic resonance spectroscopy studies, which showed that the brain GABA level in depressive patients was lower than that in healthy controls, but no difference was found in depressive patients in remission [39]. Several postmortem studies have shown decreased levels of the GABA synthase glutamic acid decarboxylase in the prefrontal cortex of patients with depression [40, 41]. It has been suggested that a functional imbalance of the GABA and glutamate systems contributes to the pathophysiology of depression, and activation of the GABA system might induce antidepressant activity, by which GABA_A_ receptor mediators α2/α3 are considered potential antidepressant candidates [42, 43]. Genetic mouse models, such as the GABA_A_ receptor mutant mouse and conditional the Gad1-knockout mouse (GABA in hippocampus and cerebral cortex decreased by 50%) and optogenetic methods have verified that depression-like behavior is induced by changing the level of GABA [44, 45].

### Neurotrophin Family

The neurotrophin family plays a key role in neuroplasticity and neurogenesis. The neurotrophic hypothesis of depression postulates that a deficit of neurotrophic support leads to neuronal atrophy, the reduction of neurogenesis, and the destruction of glia support, while antidepressants attenuate or reverse these pathophysiological processes [46]. Among them, the most widely accepted hypothesis involves brain-derived neurotrophic factor (BDNF). This was initially triggered by evidence that stress reduces the BDNF levels in the animal brain, while antidepressants rescue or attenuate this reduction [47, 48], and agents involved in the BDNF system have been reported to exert antidepressant-like effects [49, 50]. In addition, mounting studies have reported that the BDNF level is decreased in the peripheral blood and at post-mortem in depressive patients, and some have reported that antidepressant treatment normalizes it [51, 52]. Furthermore, some evidence also showed that the interaction of BDNF and its receptor gene is associated with treatment-resistant depression [15].

Recent studies reported that depressed patients have a lower level of the pro-domain of BDNF (BDNF pro-peptide) than controls. This is located presynaptically and promotes long-term depression in the hippocampus, suggesting that it is a promising synaptic regulator [53].

### Neuroinflammation

The immune-inflammation hypothesis has attracted much attention, suggesting that the interactions between inflammatory pathways and neural circuits and neurotransmitters are involved in the pathogenesis and pathophysiological processes of depression. Early evidence found that patients with autoimmune or infectious diseases are more likely to develop depression than the general population [54]. In addition, individuals without depression may display depressive symptoms after treatment with cytokines or cytokine inducers, while antidepressants relieve these symptoms [55, 56]. There is a complex interaction between the peripheral and central immune systems. Previous evidence suggested that peripheral inflammation/infection may spread to the central nervous system in some way and cause a neuroimmune response [55, 57]: (1) Some cytokines produced in the peripheral immune response, such as IL-6 and IL-1 β, can leak into the brain through the blood-brain barrier (BBB). (2) Cytokines entering the central nervous system act directly on astrocytes, small stromal cells, and neurons. (3) Some peripheral immune cells can cross the BBB through specific transporters, such as monocytes. (4) Cytokines and chemokines in the circulation activate the central nervous system by regulating the surface receptors of astrocytes and endothelial cells at the BBB. (5) As an intermediary pathway, the immune inflammatory response transmits peripheral danger signals to the center, amplifies the signals, and shows the external phenotype of depressive behavior associated with stress/trauma/infection. (6) Cytokines and chemokines may act directly on neurons, change their plasticity and promote depression-like behavior.

Patients with depression show the core feature of the immune-inflammatory response, that is, increased concentrations of pro-inflammatory cytokines and their receptors, chemokines, and soluble adhesion molecules in peripheral blood and cerebrospinal fluid [58–60]. Peripheral immune-inflammatory response markers not only change the immune activation state in the brain that affects explicit behavior, but also can be used as an evaluation index or biological index of antidepressant therapy [61, 62]. Li *et al*. showed that the level of TNF-α in patients with depression prior to treatment was higher than that in healthy controls. After treatment with venlafaxine, the level of TNF-α in patients with depression decreased significantly, and the level of TNF-α in the effective group decreased more [63]. A recent meta-analysis of 1,517 patients found that antidepressants significantly reduced peripheral IL-6, TNF-α, IL-10, and CCL-2, suggesting that antidepressants reduce markers of peripheral inflammatory factors [64]. Recently, Syed *et al*. also confirmed that untreated patients with depression had higher levels of inflammatory markers and increased levels of anti-inflammatory cytokines after antidepressant treatment, while increased levels of pro-inflammatory cytokines were found in non-responders [62]. Clinical studies have also found that anti-inflammatory cytokines, such as monoclonal antibodies and other cytokine inhibitors, may play an antidepressant role by blocking cytokines. The imbalance of pro-inflammatory and anti-inflammatory cytokines may be involved in the pathophysiological process of depression.

In addition, a recent study showed that microglia contribute to neuronal plasticity and neuroimmune interaction that are involved in the pathophysiology of depression [65]. When activated microglia promote inflammation, especially the excessive production of pro-inflammatory factors and cytotoxins in the central nervous system, depression-like behavior can gradually develop [65, 66]. However, microglia change polarization as two types under different inflammatory states, regulating the balance of pro- and anti-inflammatory factors. These two types are M1 and M2 microglia; the former produces large number of pro-inflammatory cytokines after activation, and the latter produces anti-inflammatory cytokines. An imbalance of M1/M2 polarization of microglia may contribute to the pathophysiology of depression [67].

### Microbiome-Gut-Brain Axis

The microbiota-gut-brain axis has recently gained more attention because of its ability to regulate brain activity. Many studies have shown that the microbiota-gut-brain axis plays an important role in regulating mood, behavior, and neuronal transmission in the brain [68, 69]. It is well established that comorbidity of depression and gastrointestinal diseases is common [70, 71]. Some antidepressants can attenuate the symptoms of patients with irritable bowel syndrome and eating disorders [72]. It has been reported that gut microbiome alterations are associated with depressive-like behaviors [73, 74], and brain function [75]. Early animal studies have shown that stress can lead to long-term changes in the diversity and composition of intestinal microflora, and is accompanied by depressive behavior [76, 77]. Interestingly, some evidence indicates that rodents exhibit depressive behavior after fecal transplants from patients with depression [74]. On the other hand, some probiotics attenuated depressive-like behavior in animal studies, [78] and had antidepressant effects on patients with depression in several double-blind, placebo-controlled clinical trials [79, 80].

The potential mechanism may be that gut microbiota can interact with the brain through a variety of pathways or systems, including the HPA axis, and the neuroendocrine, autonomic, and neuroimmune systems [81]. For example, recent evidence demonstrated that gut microbiota can affect the levels of neurotransmitters in the gut and brain, including serotonin, dopamine, noradrenalin, glutamate, and GABA [82]. In addition, recent studies showed that changes in gut microbiota can also impair the gut barrier and promote higher levels of peripheral inflammatory cytokines [83, 84]. Although recent research in this area has made significant progress, more clinical trials are needed to determine whether probiotics have any effect on the treatment of depression and what the potential underlying mechanisms are.

### Other Systems and Pathways

There is no doubt that several other systems or pathways are also involved in the pathophysiology of depression, such as oxidant-antioxidant imbalance [85], mitochondrial dysfunction [86, 87], and circadian rhythm-related genes [88], especially their critical interactions (*e.g.* interaction between the HPA and mitochondrial metabolism [89, 90], and the reciprocal interaction between oxidative stress and inflammation [2, 85]). The pathogenesis of depression is complex and all the hypotheses should be integrated to consider the many interactions between various systems and pathways.

## Advances in Various Kinds of Research on Depressive Disorder

Genetic, molecular, and neuroimaging studies continue to increase our understanding of the neurobiological basis of depression. However, it is still not clear to what extent the results of neurobiological studies can help improve the clinical and functional prognosis of patients. Therefore, over the past 10 years, the neurobiological study of depression has become an important measure to understand the pathophysiological mechanism and guide the treatment of depression.

### Genetic Studies

Previous twin and adoption studies have indicated that depression has relatively low rate of heritability at 37% [91]. In addition, environmental factors such as stressful events are also involved in the pathogenesis of depression. Furthermore, complex psychiatric disorders, especially depression, are considered to be polygenic effects that interact with environmental factors [13]. Therefore, reliable identification of single causative genes for depression has proved to be challenging. The first genome-wide association studies (GWAS) for depression was published in 2009, and included 1,738 patients and 1,802 controls [92, 93]. Although many subsequent GWASs have determined susceptible genes in the past decade, the impact of individual genes is so small that few results can be replicated [94, 95]. So far, it is widely accepted that specific single genetic mutations may play minor and marginal roles in complex polygenic depression. Another major recognition in GWASs over the past decade is that prevalent candidate genes are usually not associated with depression. Further, the inconsistent results may also be due to the heterogeneity and polygenic nature of genetic and non-genetic risk factors for depression as well as the heterogeneity of depression subtypes [95, 96]. Therefore, to date, the quality of research has been improved in two aspects: (1) the sample size has been maximized by combining the data of different evaluation models; and (2) more homogenous subtypes of depression have been selected to reduce phenotypic heterogeneity [97]. Levinson *et al*. pointed out that more than 75,000 to 100,000 cases should be considered to detect multiple depression associations [95]. Subsequently, several recent GWASs with larger sample sizes have been conducted. For example, Okbay *et al*. identified two loci associated with depression and replicated them in separate depression samples [98]. Wray *et al*. also found 44 risk loci associated with depression based on 135,458 cases and 344,901 controls [99]. A recent GWAS of 807,553 individuals with depression reported that 102 independent variants were associated with depression; these were involved in synaptic structure and neural transmission, and were verified in a further 1,507,153 individuals [100]. However, even with enough samples, GWASs still face severe challenges. A GWAS only marks the region of the genome and is not directly related to the potential biological function. In addition, a genetic association with the indicative phenotype of depression may only be part of many pathogenic pathways, or due to the indirect influence of intermediate traits in the causal pathway on the final result [101].

Given the diversity of findings, epigenetic factors are now being investigated. Recent studies indicated that epigenetic mechanisms may be the potential causes of "loss of heritability" in GWASs of depression. Over the past decade, a promising discovery has been that the effects of genetic information can be directly influenced by environment factors, and several specific genes are activated by environmental aspects. This process is described as interactions between genes and the environment, which is identified by the epigenetic mechanism. Environmental stressors cause alterations in gene expression in the brain, which may cause abnormal neuronal plasticity in areas related to the pathogenesis of the disease. Epigenetic events alter the structure of chromatin, thereby regulating gene expression involved in neuronal plasticity, stress behavior, depressive behavior, and antidepressant responses, including DNA methylation, histone acetylation, and the role of non-coding RNA. These new mechanisms of trans-generational transmission of epigenetic markers are considered a supplement to orthodox genetic heredity, providing the possibility for the discovery of new treatments for depression [102, 103]. Recent studies imply that life experiences, including stress and enrichment, may affect cellular and molecular signaling pathways in sperm and influence the behavioral and physiological phenotypes of offspring in gender-specific patterns, which may also play an important role in the development of depression [103].

### Brain Imaging and Neuroimaging Studies

Neuroimaging, including magnetic resonance imaging (MRI) and molecular imaging, provides a non-invasive technique for determining the underlying etiology and individualized treatment for depression. MRI can provide important data on brain structure, function, networks, and metabolism in patients with depression; it includes structural MRI (sMRI), functional MRI (fMRI), diffusion tensor imaging, and magnetic resonance spectroscopy.

Previous sMRI studies have found damaged gray matter in depression-associated brain areas, including the frontal lobe, anterior cingulate gyrus, hippocampus, putamen, thalamus, and amygdala. sMRI focuses on the thickness of gray matter and brain morphology [104, 105]. A recent meta-analysis of 2,702 elderly patients with depression and 11,165 controls demonstrated that the volumes of the whole brain and hippocampus of patients with depression were lower than those of the control group [106]. Some evidence also showed that the hippocampal volume in depressive patients was lower than that of controls, and increased after treatment with antidepressants [107] and electroconvulsive therapy (ECT) [108], suggesting that the hippocampal volume plays a critical role in the development, treatment response, and clinical prognosis of depression. A recent study also reported that ECT increased the volume of the right hippocampus, amygdala, and putamen in patients with treatment-resistant depression [109]. In addition, postmortem research supported the MRI study showing that dentate gyrus volume was decreased in drug-naive patients with depression compared to healthy controls, and was potentially reversed by treatment with antidepressants [110].

Diffusion tensor imaging detects the microstructure of the white matter, which has been reported impaired in patients with depression [111]. A recent meta-analysis that included first-episode and drug-naïve depressive patients showed that the decrease in fractional anisotropy was negatively associated with illness duration and clinical severity [112].

fMRI, including resting-state and task-based fMRI, can divide the brain into self-related regions, such as the anterior cingulate cortex, posterior cingulate cortex, medial prefrontal cortex, precuneus, and dorsomedial thalamus. Many previous studies have shown the disturbance of several brain areas and intrinsic neural networks in patients with depression which could be rescued by antidepressants [113–116]. Further, some evidence also showed an association between brain network dysfunction and the clinical correlates of patients with depression, including clinical symptoms [117] and the response to antidepressants [118, 119], ECT [120, 121], and repetitive transcranial magnetic stimulation [122].

It is worth noting that brain imaging provides new insights into the large-scale brain circuits that underlie the pathophysiology of depressive disorder. In such studies, large-scale circuits are often referred to as “networks”. There is evidence that a variety of circuits are involved in the mechanisms of depressive disorder, including disruption of the default mode, salience, affective, reward, attention, and cognitive control circuits [123]. Over the past decade, the study of intra-circuit and inter-circuit connectivity dysfunctions in depression has escalated, in part due to advances in precision imaging and analysis techniques [124]. Circuit dysfunction is a potential biomarker to guide psychopharmacological treatment. For example, Williams *et al*. found that hyper-activation of the amygdala is associated with a negative phenotype that can predict the response to antidepressants [125]. Hou *et al*. showed that the baseline characteristics of the reward circuit predict early antidepressant responses [126].

Molecular imaging studies, including single photon emission computed tomography and positron emission tomography, focus on metabolic aspects such as amino-acids, neurotransmitters, glucose, and lipids at the cellular level in patients with depression. A recent meta-analysis examined glucose metabolism and found that glucose uptake dysfunction in different brain regions predicts the treatment response [127].

The most important and promising studies were conducted by the ENIGMA (Enhancing NeuroImaging Genetics through Meta Analysis) Consortium, which investigated the human brain across 43 countries. The ENIGMA-MDD Working Group was launched in 2012 to detect the structural and functional changes associated with MDD reliably and replicate them in various samples around the world [128]. So far, the ENIGMA-MDD Working Group has collected data from 4,372 MDD patients and 9,788 healthy controls across 14 countries, including 45 cohorts [128]. Their findings to date are shown in Table 1 [128–137].

**Table 1.** Findings of the ENIGMA (Enhancing NeuroImaging Genetics through Meta Analysis) Consortium.

Medicine type	Samples	Methods	Region	Method of analysis	Main outcomes
Schmaal *et al.* 2016 [142]	1,728 MDD patients and 7,199 controls	sMRI	Subcortical volumes	Meta-analysis	Hippocampal volume was lower in MDD than controls. Early age of onset (≤21) was associated with a smaller hippocampus and larger lateral ventricles in MDD patients
Schmaal *et al.* 2017 [143]	2,148 MDD patients and 7,957 healthy controls	sMRI	Cortical thickness and surface region	Meta-analysis	Gray matter of orbital prefrontal lobe, anterior cingulate gyrus, posterior cingulate gyrus, insular lobe, and temporal lobe in adult MDD patients was thinner than that in the control group. Total surface areas and frontal lobe areas, primary and higher-order visual, somatosensory, and motor areas in adolescents with MDD were thinner than that in the control group
Frodl *et al.* 2017 [144]	958 MDD patients and 2,078 healthy controls	sMRI	Subcortical volumes	Mega-analysis	Significant interactions among childhood adversity, MDD diagnosis, sex, and region. Childhood adversity was associated with lower caudate volumes in female participants
Renteria *et al.* 2017 [145]	451 MDD patients with suicide, 650 MDD patients without suicide, and 1,996 healthy controls	sMRI	Subcortical grey matter, lateral ventricle, and total intracranial volume	Meta-analysis	MDD with suicide (reported suicidal attempts or plans) had a smaller total intracranial volume and a 2.87% reduction in volume than controls. There was no difference between MDD patients with suicidal symptoms and controls. There was no difference in brain volume between MDD patients with and without suicidal symptoms
Tozzi *et al.* 2019 [146]	1,284 MDD patients and 2,588 healthy controls.	sMRI	Cortical thickness and surface region	Mega-analysis	Childhood maltreatment severity was associated with decreased thickness in the supramarginal gyrus and banks of the superior temporal sulcus, and decreased surface area of the middle temporal lobe, as well as with higher cortical thickness of the rostral anterior cingulate cortex in male participants
de Kovel *et al.* 2019 [147]	Cortical regions: 2,256 MDD patients and 3,504 healthy controls. Subcortical regions: 2,540 MDD patients and 4,230 healthy controls	sMRI	Subcortical volumes, cortical thickness, and surface region	Mega-analysis	No differences in the laterality of cortical thickness or subcortical volumes or surface area in MDD patients and controls
Ho *et al.* 2020 [148]	1,781 MDD patients and 2,953 healthy controls	sMRI	Shape metrics in thickness and surface region of subcortical structures	Meta-analysis	Thickness and surface region of the subiculum, CA1 of the hippocampus, and basolateral amygdala were lower in MDD patients with adolescent-onset MDD (≤21 years). Thickness and surface region of hippocampal CA1 and basolateral amygdala were lower in patients with recurrent MDD than in those with first-episode MDD
Han *et al.* 2020 [149]	2,675 MDD patients and 4,314 healthy controls	sMRI	Subcortical volumes, cortical thickness, and surface region	Mega-analysis	MDD patients had a higher brain predicted age difference of 1.08 years than controls, which indicated patterns of age-related structural abnormalities in MDD
Van Velzen *et al.* 2020 [150]	1,305 MDD patients and 1,602 healthy controls	DTI	White matter anisotropy and diffusivity	Meta-analysis	Adult MDD patients had slightly but generally lower fractional anisotropy in 16 of the 25 white matter regions of interest than controls, especially the largest differences were in the corpus callosum and corona radiata. Adult MDD patients had widespread higher radial diffusivity than controls. However, there was no difference between adolescents with MDD and adolescent controls

## Objective Index for Diagnosis of MDD

To date, the clinical diagnosis of depression is subjectively based on interviews according to diagnostic criteria (*e.g.* International Classification of Diseases and Diagnostic and Statistical Manual diagnostic systems) and the severity of clinical symptoms are assessed by questionnaires, although patients may experience considerable differences in symptoms and subtypes [138]. Meanwhile, biomarkers including genetics, epigenetics, peripheral gene and protein expression, and neuroimaging markers may provide a promising supplement for the development of the objective diagnosis of MDD, [139–141]. However, the development of reliable diagnosis for MDD using biomarkers is still difficult and elusive, and all methods based on a single marker are insufficiently specific and sensitive for clinical use [142]. Papakostas *et al*. showed that a multi-assay, serum-based test including nine peripheral biomarkers (soluble tumor necrosis factor alpha receptor type II, resistin, prolactin, myeloperoxidase, epidermal growth factor, BDNF, alpha1 antitrypsin, apolipoprotein CIII, brain-derived neurotrophic factor, and cortisol) yielded a specificity of 81.3% and a sensitivity of 91.7% [142]. However, the sample size was relatively small and no other studies have yet validated their results. Therefore, further studies are needed to identify biomarker models that integrate all biological variables and clinical features to improve the specificity and sensitivity of diagnosis for MDD.

## Management of Depression

The treatment strategies for depression consist of pharmacological treatment and non-pharmacological treatments including psychotherapy, ECT [98], and transcranial magnetic stimulation. As psychotherapy has been shown to have effects on depression including attenuating depressive symptoms and improving the quality of life [143, 144]; several practice guidelines are increasingly recommending psychotherapy as a monotherapy or in combination with antidepressants [145, 146].

### Current Antidepressant Treatment

Antidepressants approved by the US Food and Drug Administration (FDA) are shown in Table 2. Due to the relatively limited understanding of the etiology and pathophysiology of depression, almost all the previous antidepressants were discovered by accident a few decades ago. Although most antidepressants are usually safe and effective, there are still some limitations, including delayed efficacy (usually 2 weeks) and side-effects that affect the treatment compliance [147]. In addition, <50% of all patients with depression show complete remission through optimized treatment, including trials of multiple drugs with and without simultaneous psychotherapy. In the past few decades, most antidepressant discoveries focused on finding faster, safer, and more selective serotonin or norepinephrine receptor targets. In addition, there is an urgent need to develop new approaches to obtain more effective, safer, and faster antidepressants. In 2019, the FDA approved two new antidepressants: Esketamine for refractory depression and Bresanolone for postpartum depression. Esmolamine, a derivative of the anesthetic drug ketamine, was approved by the FDA for the treatment of refractory depression, based on a large number of preliminary clinical studies [148]. For example, several randomized controlled trials and meta-analysis studies showed the efficacy and safety of Esketamine in depression or treatment-resistant depression [26, 149, 150]. Although both are groundbreaking new interventions for these debilitating diseases and both are approved for use only under medical supervision, there are still concerns about potential misuse and problems in the evaluation of mental disorders [151].

**Table 2.** Antidepressants approved across the world.

Medicine type	Example	Psychopharmacological mechanism of effect
Tricyclic antidepressants	Amitriptyline, Maprotiline, Nortriptyline, Protriptyline, Trimipramine, Desipramine, Doxepin, Imipramine	Non-selective inhibitors of monoamines reuptake, including serotonin, dopamine, and norepinephrine
MAO inhibitors	Selegiline, Tranylcypromine, Phenylzine, Isocarboxazid	Inhibitors of enzymes (MAO-B, MAO-A and MAO-B)
Selective serotonin reuptake inhibitors	Fluoxetine, Sertraline, Paroxetine, Fluvoxamine, Citalopram, Escitalopram	Selective serotonin reuptake inhibitors
Serotonin-norepinephrine reuptake inhibitors	Venlafaxine, Desvenlafaxine, Duloxetine	Serotonin and norepinephrine reuptake inhibitors
Noradrenergic and specific serotonergic modulator	Mirtazapine	Noradrenergic and specific serotonergic antidepressant
MT1/MT2 agonist and 5-HT2C antagonist	Agomelatine	Antagonism at MT1 and MT2 and antagonism at 5-HT2C receptors
Multimodal antidepressant	Vortioxetine	Combination of two pharmacological modes of action: reuptake inhibition and receptor activity across five pharmacological targets
Norepinephrine-dopamine reuptake inhibitor	Bupropion	Releasing agent of dopamine and norepinephrine
Serotonin modulators	Trazodone, Nefazodone	Serotonin 5-HT2A antagonists
Serotonin reuptake inhibitor and 5-HT1A-receptor partial agonist	Vilazodone	Inhibiting serotonin reuptake and acts as a partial agonist at the 5-HT1A receptor
Non-competitive N-methyl-D-aspartate (NMDA) receptor antagonist	Esketamine	Non-competitive NMDA receptor antagonist
Neurosteroid	Bresanolone	Positive allosteric modulator of the GABAA receptor

To date, although several potential drugs have not yet been approved by the FDA, they are key milestones in the development of antidepressants that may be modified and used clinically in the future, such as compounds containing dextromethorphan (a non-selective NMDAR antago–nist), sarcosine (N-methylglycine, a glycine reuptake inhibitor), AMPAR modulators, and mGluR modulators [152].

### Neuromodulation Therapy

Neuromodulation therapy acts through magnetic pulse, micro-current, or neural feedback technology within the treatment dose, acting on the central or peripheral nervous system to regulate the excitatory/inhibitory activity to reduce or attenuate the symptoms of the disease.

ECT is one of most effective treatments for depression, with the implementation of safer equipment and advancement of techniques such as modified ECT [153]. Mounting evidence from randomized controlled trial (RCT) and meta-analysis studies has shown that rTMS can treat depressive patients with safety [154]. Other promising treatments for depression have emerged, such as transcranial direct current stimulation (tDCS) [155], transcranial alternating current stimulation (tACS)[156], vagal nerve stimulation [157], deep brain stimulation [158] , and light therapy [159], but some of them are still experimental to some extent and have not been widely used. For example, compared to tDCS, tACS displays less sensory experience and adverse reactions with weak electrical current in a sine-wave pattern, but the evidence for the efficacy of tACS in the treatment of depression is still limited [160]. Alexander *et al*. recently demonstrated that there was no difference in efficacy among different treatments (sham, 10-Hz and 40-Hz tACS). However, only the 10-Hz tACS group had more responders than the sham and 40-Hz tACS groups at week 2 [156]. Further RCT studies are needed to verify the efficacy of tACS. In addition, the mechanism of the effect of neuromodulation therapy on depression needs to be further investigated.

### Precision Medicine for Depression

Optimizing the treatment strategy is an effective way to improve the therapeutic effect on depression. However, each individual with depression may react very differently to different treatments. Therefore, this raises the question of personalized treatment, that is, which patients are suitable for which treatment. Over the past decade, psychiatrists and psychologists have focused on individual biomarkers and clinical characteristics to predict the efficiency of antidepressants and psychotherapies, including genetics, peripheral protein expression, electrophysiology, neuroimaging, neurocognitive performance, developmental trauma, and personality [161]. For example, Bradley *et al*. recently conducted a 12-week RCT, which demonstrated that the response rate and remission rates of the pharmacogenetic guidance group were significantly higher than those of the non-pharmacogenetic guidance group [162].

Subsequently, Greden *et al*. conducted an 8-week RCT of Genomics Used to Improve Depression Decisions (GUIDED) on 1,167 MDD patients and demonstrated that although there was no difference in symptom improvement between the pharmacogenomics-guided and non- pharmacogenomics-guided groups, the response rate and remission rate of the pharmacogenomics-guided group increased significantly [163].

A recent meta-analysis has shown that the baseline default mode network connectivity in patients with depression can predict the clinical responses to treatments including cognitive behavioral therapy, pharmacotherapy, ECT, rTMS, and transcutaneous vagus nerve stimulation [164]. However, so far, the biomarkers that predict treatment response at the individual level have not been well applied in the clinic, and there is still a lot of work to be conducted in the future.

## Future Perspectives

Although considerable progress has been made in the study of depression during a past decade, the heterogeneity of the disease, the effectiveness of treatment, and the gap in translational medicine are critical challenges. The main dilemma is that our understanding of the etiology and pathophysiology of depression is inadequate, so our understanding of depression is not deep enough to develop more effective treatment. Animal models still cannot fully simulate this heterogeneous and complex mental disorder. Therefore, how to effectively match the indicators measured in animals with those measured in genetic research or the development of new antidepressants is another important challenge.
